# Sesamol Loaded Albumin Nanoparticles: A Boosted Protective Property in Animal Models of Oxidative Stress

**DOI:** 10.3390/ph15060733

**Published:** 2022-06-10

**Authors:** Sara Zaher, Mahmoud E. Soliman, Mahmoud Elsabahy, Rania M. Hathout

**Affiliations:** 1 Assiut International Center of Nanomedicine, Al-Rajhy Liver Hospital, Assiut University, Assiut 71515, Egypt; sarazaher914@gmail.com; 2 Department of Pharmaceutics and Industrial Pharmacy, Faculty of Pharmacy, Ain Shams University, Cairo 11566, Egypt; mahmoud.e.soliman@pharma.asu.edu.eg; 3 Pharm D Program, Egypt-Japan University of Science and Technology (EJUST), New Borg El Arab, Alexandria 21934, Egypt; 4 School of Biotechnology and Science Academy, Badr University in Cairo, Badr City, Cairo 11829, Egypt; mahmoud.elsabahy@buc.edu.eg

**Keywords:** sesamol, albumin nanoparticles, doxorubicin, oxidative stress, antioxidants

## Abstract

The current study evaluated the ability of sesamol-loaded albumin nanoparticles to impart protection against oxidative stress induced by anthracyclines in comparison to the free drug. Albumin nanoparticles were prepared via the desolvation technique and then freeze-dried with the cryoprotectant, trehalose. Albumin concentration, pH, and type of desolvating agent were assessed as determining factors for successful albumin nanoparticle fabrication. The optimal nanoparticles were spherical in shape, and they had an average particle diameter of 127.24 ± 2.12 nm with a sesamol payload of 96.89 ± 2.4 μg/mg. The drug cellular protection was tested on rat hepatocytes pretreated with 1 µM doxorubicin, which showed a 1.2-fold higher protective activity than the free sesamol. In a pharmacokinetic study, the loading of a drug onto nanoparticles resulted in a longer half-life and mean residence time, as compared to the free drug. Furthermore, in vivo efficacy and biochemical assessment of lipid peroxidation, cardiac biomarkers, and liver enzymes were significantly ameliorated after administration of the sesamol-loaded albumin nanoparticles. The biochemical assessments were also corroborated with the histopathological examination data. Sesamol-loaded albumin nanoparticles, prepared under controlled conditions, may provide an enhanced protective effect against off-target doxorubicin toxicity.

## 1. Introduction

Excessive free radical generation and cellular oxidative stress are implicated as a causative or as an adjuvant of various pathologies, such as hepatic fibrosis, pulmonary inflammation, cardiomyopathy, diabetic complications, renal disease, brain aging, and neurodegenerative conditions [1,2,3]. Several chemotherapeutic agents are a potential source of free radicals and reactive species, which impede their therapeutic use [4]. Free radicals are involved in cellular respiration, cell growth regulation, intracellular signaling, and biomolecule synthesis [5].

Doxorubicin (DOX), a secondary metabolite of *Streptomyces peucetius* that belongs to the family of anthracycline drugs, is a potent antineoplastic agent that is widely used to treat different adult and pediatric tumors, including solid tumors, leukemias, breast cancer, and Hodgkin’s and non-Hodgkin’s lymphoma. It works by multiple cytotoxic pathways and the most potent is targeting topoisomerase II α, which is highly expressed in cancer cells, to inhibit DNA replication [6]. Unfortunately, DOX is metabolized by cytochrome P450 and flavin-monooxygenase enzymes to its semiquinone form, which interacts with oxygen molecules to generate reactive oxygen species (ROS) [4,7]. Additionally, DOX molecules interact with iron (Fe^2+^) forming a free radical complex, which is usually involved in the production of hydroxyl radical (HO•). Excessive ROS generation triggers the lipid peroxidation and the depletion of endogenous antioxidant enzymes, resulting in mitochondrial dysfunction, cellular injury, and multi-organ toxicity [8]. Hence, searching for a protective agent remains a challenge [9].

Currently, there is great interest toward the combination of natural antioxidants such as, curcumin, silibinin, or sesamol (SML), and conventional antineoplastic agents such as, cisplatin, and doxorubicin as prospects for chemotherapy to avoid harmful [10] effects, which can be toxic to healthy cells [11,12,13,14]. Sesamol (3,4-methylenedioxy phenol, SML), a phenolic antioxidant agent, is a major component of sesame seed oil. SML has a potent antioxidant, anti-inflammatory, and free radical scavenging activity [10,15].

Several researchers have demonstrated the protective effect of SML on oxidative stress caused by DOX. It has been reported to possess various physiological activities as a hepatoprotective, cardioprotective, antiatherogenic, and anti-aging agent by attenuating oxidative stress and systemic inflammation [16]. However, SML has poor intracellular bioavailability (F_ic_) due to its hydrophilic nature [17]. SML also suffers from rapid clearance that is associated with the appearance of its metabolites such as sesamol sulphate/glucuronide within four hours [18,19]. Hence, a sustained release formulation can provide a controlled and a steady release manner to maintain SML plasma concentration that is stable for a prolonged time. Albumin nanoparticles that can be internalized and engulfed by cells may provide sustained drug release and prolonged efficacy.

Human serum albumin (HSA) is an endogenous, non-toxic, biodegradable, non-immunogenic, water-soluble polymer (Generally Regarded As Safe (GRAS)) that possesses serum stability and a long half-life [20]. In addition, HSA provides sites for the binding of various polar and nonpolar drugs. Further, albumin-based nanoparticles (ANPs) play a vital role in passive targeting, and they accumulate in solid tumors and inflamed tissues due to the enhanced permeability and retention effect [21]. The aforementioned preferable properties of albumin form the rationale for developing albumin-based drug delivery systems [20]. Clinically, several drugs have been loaded onto ANPs and approved for use as DOX, methotrexate, and paclitaxel.

It is worth noting that HSA possesses direct and indirect antioxidant effects. It works directly as a free radical scavenger as the free thiol groups in the Cys-34 residue are able to trap different types of ROS and six methionine groups, which are easily oxidized. Moreover, HSA can act indirectly by binding bilirubin that inhibit lipid peroxidation and free metals such as iron and copper, which catalyze aggressive ROS formation [22].

The work in this study comprised the fabrication of a series of SML-loaded ANPs (SML-ANPs) formulations adopting the desolvation technique. A systematic optimization concerning the influence of albumin concentration, pH of the medium, and the type of desolvation reagent on the physicochemical characteristics of nanoparticles was carried out to allow preparation of a colloidal system with well-defined physicochemical characteristics. In vitro and in vivo assessments were carried out in animal models of oxidative stress induced by DOX.

## 2. Results

### 2.1. Characterization of SML-ANPs and Statistical Modeling

**Figure 1:**
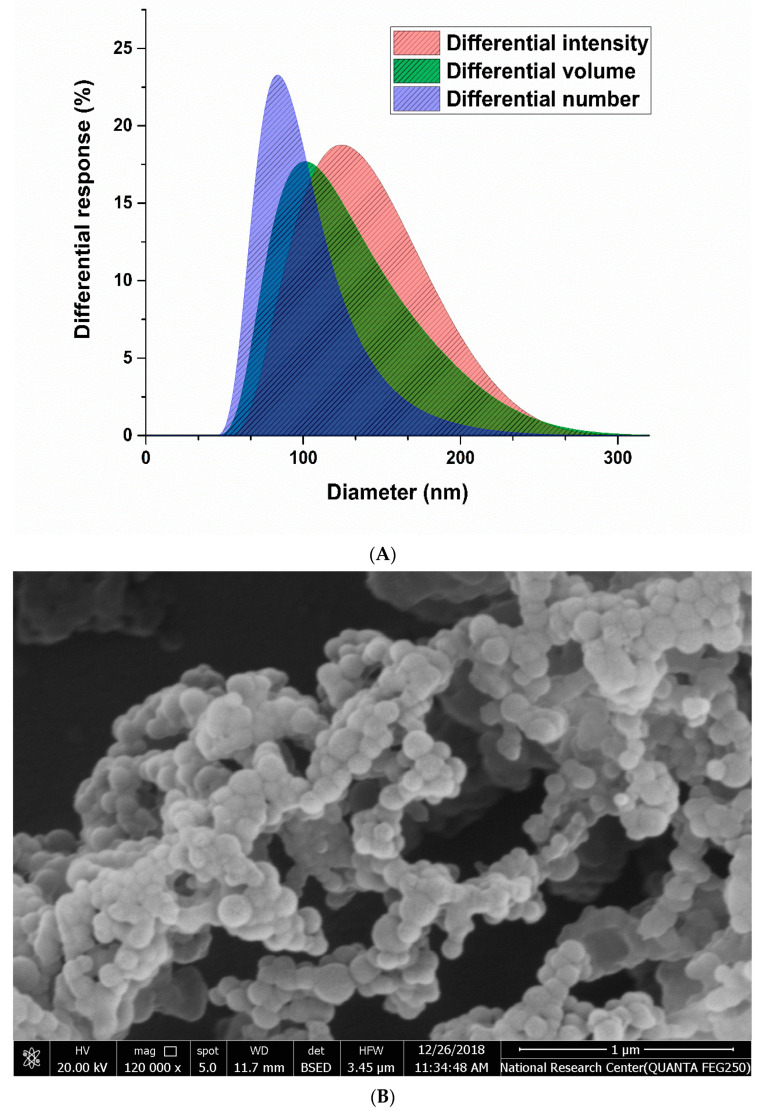
Size distribution histograms, the intensity-, volume-, and number-averaged hydrodynamic diameter histograms of a selected SML-ANPs (**A**), scanning electron micrograph (**B**).

The desolvation technique allowed fabrication of homogenous SML-ANPs with a drug loading capacity ranging from 14.8 to 116.7 µg/mg HSA. All the prepared formulations showed particle diameters ranging from 111.5 to 367.1 nm, with an acceptable PDI < 0.3 and zeta potential. The morphological appearance using SEM imaging revealed that the particles have almost a regular spherical shape with a smooth surface, as shown in Figure 1.

**Figure 2:**
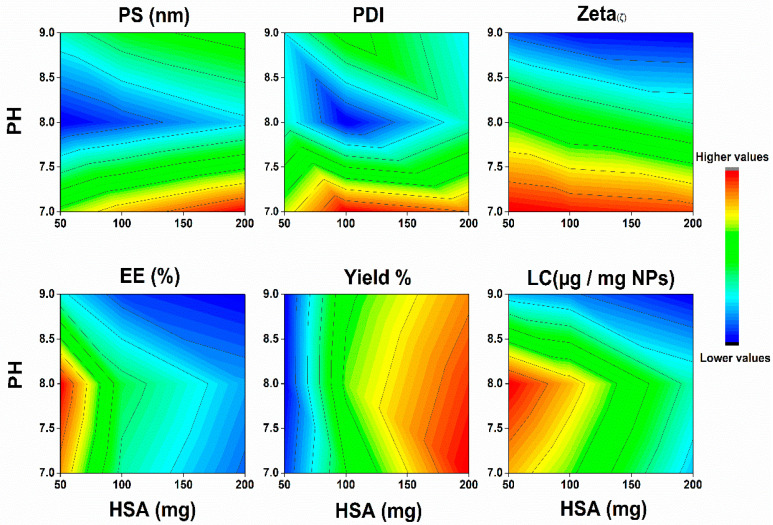
Contour plots generated by the D-optimal design demonstrating the effect of albumin concentration and aqueous medium pH using ethanol acetone as a desolvating agent on the albumin nanoparticles diameter (PS), polydispersity index (PDI), Zeta potential, sesamol encapsulation efficiency (EE%), albumin nanoparticles percent yield, and sesamol loading in albumin nanoparticles (LC).

Figure 2 summarizes the effect of the three tested variables on the fabrication of SML-ANPS. Although the increase in pH had a fluctuating effect on the particle size and the loading capacity, it was observed to cause a marked increase in the negative charge of the produced ANPS. However, optimizing HSA mass to get small monodisperse ANPS with a high loading capacity was more significant (Supplementary material, Table S1). The D-optimal design reveals the importance of each of the variables, and it jointly tests their interactions (Supplementary material, Table S2). Each response was fitted to the suggested model generated by the optimal design. The generated equations according to the DOD (Equations (S9)–(S14)) are mentioned in the supplementary file [23,24].

Multiple regression analysis of the results of the measured responses was performed, as shown in Table 1. The R2 values represent the amount of variation around the mean explained by the model. Most of the presented models were significant (*p* value < 0.001), with a good predictability (R^2^ > 0.94), demonstrating fittingness of the models except the PDI model which was insignificant. An ANOVA test was used to verify the adequacy of these models. The term “adequate precision” is a measure of the range of a predicted response relative to its associated error, which measures the signal-to-noise ratio [25]. Most of the models showed a ratio higher than 4.0, which implies an adequate signal that can navigate the design space using that model [26]. The results showed that changing the three tested factors (HSA concentration, pH, and changing type of desolvating agent) had a statistically significant impact on the physicochemical properties of the prepared SML-ANPs. The Predicted R^2^ was in reasonable agreement with the Adjusted R^2^; i.e., the difference was less than 0.2, which showed a strong correlation between the independent parameters and the responses.

Using Design-Expert software^®^ and by applying the D-optimal design desirability function, the optimum conditions for SML-ANPs fabrications were 131 mg/mL concentrations of HSA, pH 8.12, and ethanol acetone mixture as a desolvating agent with a desirability rate of 0.734. Moreover, the optimum formulation according to the predetermined parameters in the design and data analysis scored 127 nm, 0.07, −26.2 δ, 87.9%, 81.92%, and 96.9 µg/mg in particles size, PDI, zeta potential, percentage encapsulated, yield percentage, and sesamol payload, respectively, which were found to be in considerable agreement with their corresponding predicted values that were obtained by the design expert software. The mean particle size measurements using DSL were in agreement with the SEM imaging observed particle size.

### 2.2. In Vitro Cumulative Release Study

**Figure 3:**
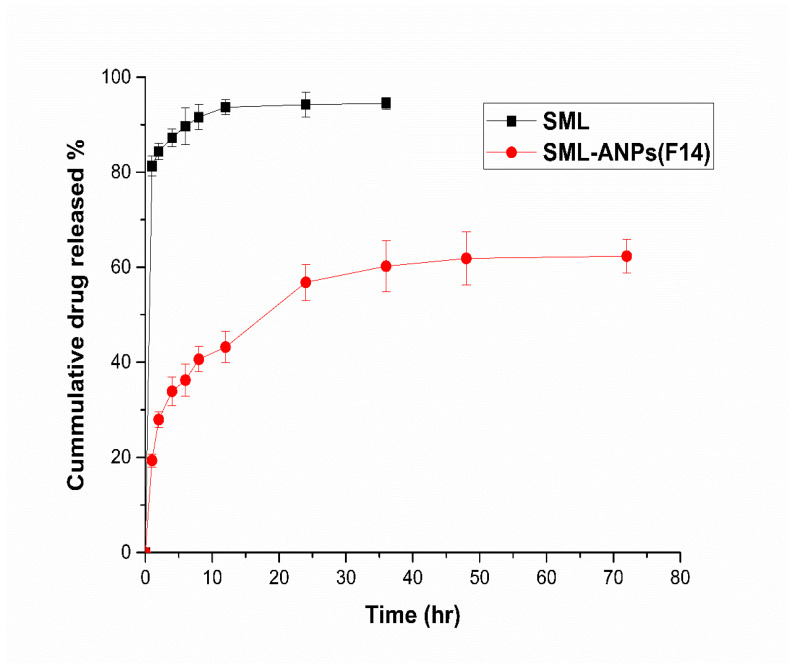
In vitro release profiles of free SML and SML-ANPs (F14). The values are presented as mean ± SD (n in each group = 3).

The in vitro release profiles of SML from the selected SML-ANPs formulations and the free drug solution are shown in Figure 3. SML was released in a biphasic manner with a sustained release subsequent to an initial burst in the first hour. The cumulative percentage of SML released over 72 h was approximately 62.3%. Table S3, summarizes the data derived from the kinetic study, where Higuchi and the Korsmeyer–Peppas model fit the SML release from SML-ANPs rather than the other modules with maximum R2 values of 0.971 and 0.985, respectively, elucidating that SML release followed the diffusion mechanism in the general manner. Besides, the value of the diffusion exponents (n) was 0.336, which corresponds to the Fickian diffusion mechanism of release [27,28].

### 2.3. Freeze Drying and Stability Studies of SML-ANPs

**Figure 4:**
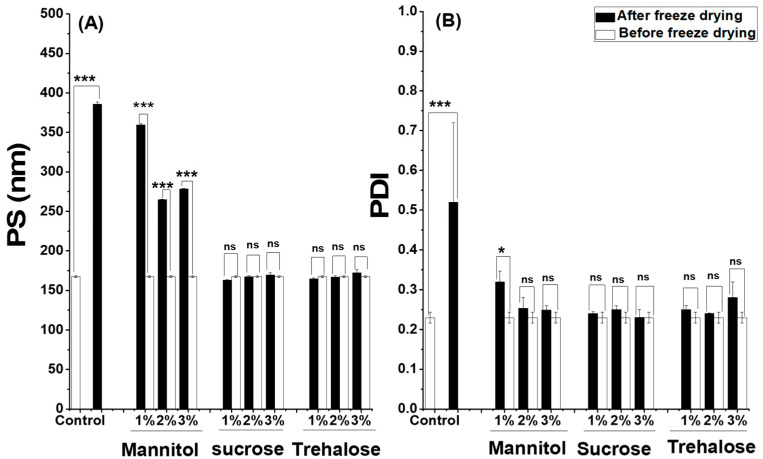
Influence of the freeze-drying process of SML-ANPs (10 mg/mL) in the presence of different excipients on (**A**) particle diameter and (**B**) polydispersity before freeze drying and after reconstitution of the samples in water (mean ± S.D.; n in each group = 3). For all results, ns presents that no significant differences were found and the differences were considered significant when *p* < 0.05, and extremely significant when *p* < 0.0001 presented by *, and ***, respectively.

The freeze-drying of the selected formulation in the absence of cryoprotectants led to a large increase in particle size reaching 386 nm and a lack of homogeneity (PDI, 0.52), as shown in Figure 4. Therefore, freeze-drying without cryoprotectants was determined as not an appropriate technique for stabilizing the suspension of SML-ANPs and enhancing its long-term stability. In the current study, the propensity of three distinct cryoprotectants (sucrose, trehalose, and mannitol) was evaluated at three concentrations of 1%, 2%, and 3% each, while retaining their original state. After reconstitution of the freeze-dried ANPs, 1% sucrose and 1% trehalose were able to maintain the original properties of SML-ANPs suspension with a low non-significant difference at the lowest tested concentration. On the other hand, mannitol showed a significant increase in particle size: 264.7 nm and a PDI of 0.3.

After reconstitution of the freeze dried SML-ANPs with different types of cryoprotectants, trehalose was able to maintain the properties of SML-ANPs, and it showed a long-term stability of up to six months with a non-significant difference (*p* ≥ 0.05). Besides, the data of the short-term stability study showed that SML-ANPs remained stable in water and biologically-relevant media, with no statistically significant changes over the 2-h incubation period in all conditions (results not shown). Regarding the shelf-life testing, three samples of freeze-dried SML-ANPs were tested weekly for 12 weeks. There was no significant change in nanoparticle diameter and PDI over the 12 weeks, except for frozen batches that became micrometric and heterogeneous (PI > 0.3) after 3 weeks of the experiment. Both types of cryoprotectants maintained their physicochemical properties at 4 °C for more than 6 months. There was no significant change in the diameter and PDI (*p* < 0.05) of the formula during the tested period for sucrose and trehalose. All of these results confirmed that sucrose and trehalose would be suitable cryoprotectants for ANPs. On the other hand, when HSA nanoparticles were not stabilized with lyophilization, the resulting carriers were found to be stable for a few weeks, then nanoparticles rapidly aggregated.

### 2.4. Pharmacokinetic Study Results

**Figure 5:**
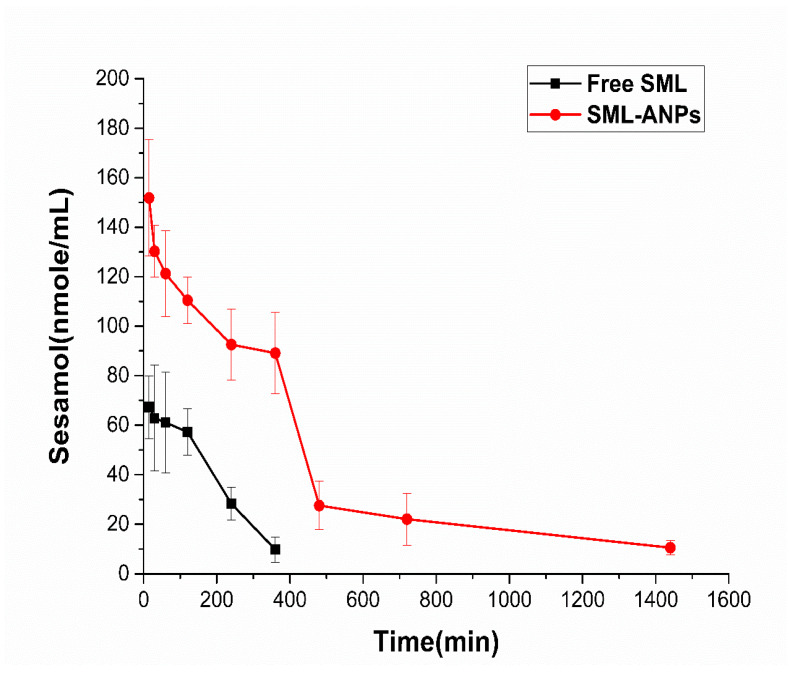
Plasma concentration–time profiles of SML after IV single dose (15 mg/kg) of SML and SML-ANPs. Values are presented as means ± SD (n in each group = 4).

Figure 5 depicts the SML plasma concentrations following intravenous administration of SML and SML-ANP at predetermined intervals. Although the clearance patterns of both groups were almost similar, a significant difference of 151.9 and 67.3 μg/mL between SML-treated and SML-ANP-treated groups, respectively, was observed early after 15 min. SML plasma concentration was 10.5 μg/mL after 24 h of an SML-ANPs injection, while SML was barely detected after 8 h in an SML-treated group. Additionally, the SML-treated group displayed a short half-life and a rapid elimination rate of 1.25 h and 0.53 L/h, respectively. As illustrated in Table 2, ANPs can act as an SML reservoir in the bloodstream, with an extended half-life and slower elimination rates of 8.9 h and 0.11 L/h, respectively. When SML-ANPs are gradually degraded, SML is slowly released into the blood stream resulting in a prolonged drug residence time in blood circulation and a higher AUC value of 8.5 h and 88.7 µg.h/L, respectively. Nevertheless, the free SML solution showed a shorter residence time and a lower AUC of 2.6 h and 19.5 µg·h/L, respectively.

The pharmacokinetic study showed that the average half-life, mean retention time, and AUC were significantly higher (*p* < 0.05) than that of the SML-treated group (Table 2), whereas the clearance rate was lower (*p* < 0.05). The above results indicate that SML-ANPs increased the systemic circulation time leading to a higher amount of SML available for cellular uptake. Moreover, maintaining the plasma SML concentration for a long time with continuous release properties was demonstrated.

### 2.5. Evaluation of SML-ANPs Hepatoprotection

**Figure 6:**
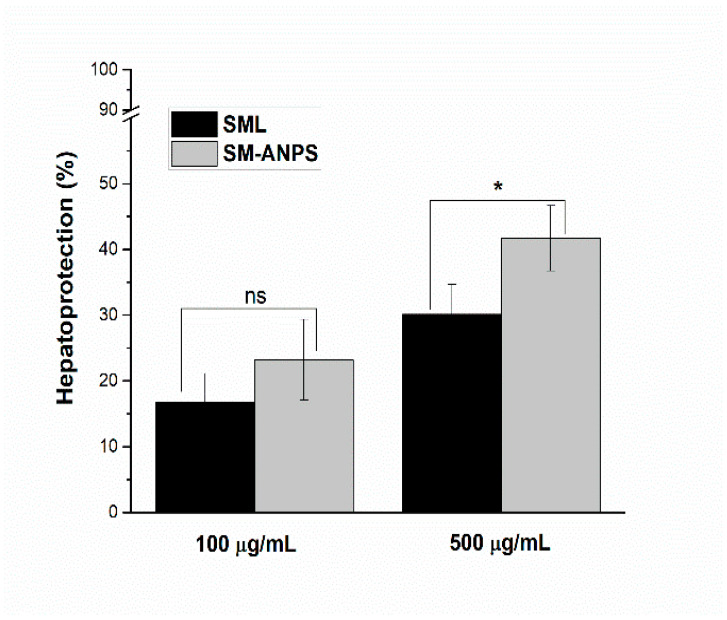
Hepatoprotective effect of SML-ANPs on rat hepatocytes in comparison to free SML solution, measured by the MTS assay. * *p* < 0.05 and ns when no significant difference was found between the SML group and the SML-ANPs group. Results presented as %hepatoprotection and expressed as the mean ± SD (n in each group = 4).

Figure 6 shows the relative cell protection of SML-ANPs at different concentrations. The results from cultivated rat hepatocytes demonstrated that cell viability was dropped to 25.2 ± 1.3% by incubation with 1 µM DOX for 24 h. Viability percentage, SML hepatoprotection, and enhanced SML-ANP protection capability were counted, as illustrated in Table S4. Treatment with SML showed a percentage of hepatoprotection at concentrations of 100 and 500 μg/mL up to 16.7 ± 4.4 and 30.1 ± 4.6, respectively. However, SML-ANPs showed an enhanced protective activity in comparison to a free SML solution at concentrations of 100 and 500 μg/mL up to 23.2 ± 6.1 and 41.7 ± 4.9 µg/mL, respectively.

### 2.6. Biochemical Assessment

**Figure 7:**
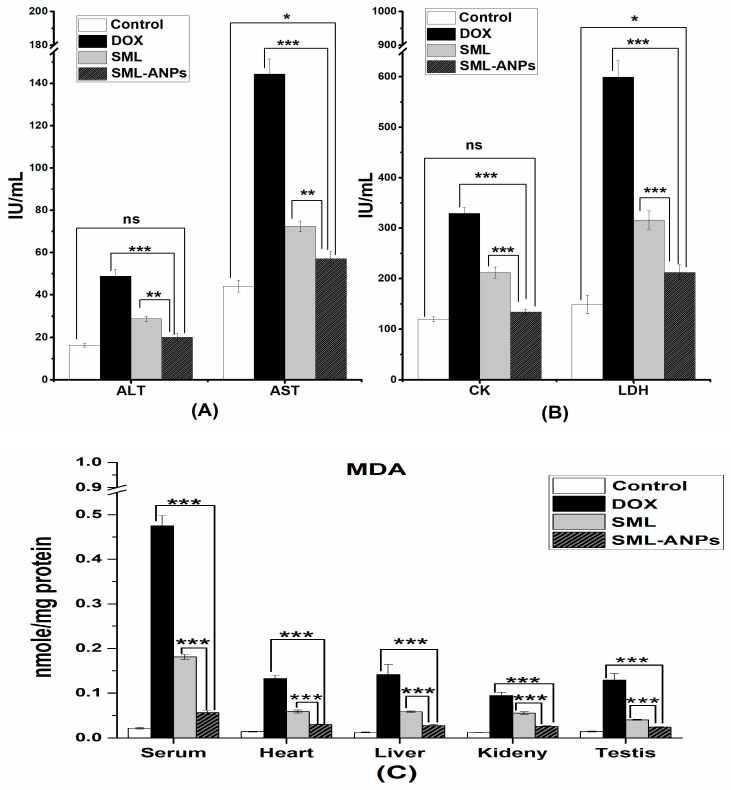
Cardiac (**A**), liver (**B**) and lipid peroxidation (**C**) biomarkers’ assessment. * *p* < 0.05, ** *p* < 0.001 and *** *p* < 0.0001 when compared to SML-ANPs group. Data are presented as means ± SE (n in each group = 8).

By cumulative i.p. injection of DOX, serum levels of CK, LDH, AST, and ALT were significantly elevated to 2.7, 4, 3.3, and 3-fold above the corresponding control values, respectively. However, the co-treatment with SML-ANPs significantly reduced the elevated serum CK and LDH by 59.3 and 64.5%, respectively, while AST and ALT were reduced by 60.5 and 59% in comparison to the corresponding values of the free SML treated group, which were 35.6, 47.2, 49.9, and 41.2%, respectively (Figure 7). These results were accompanied by a significant increase in the MDA levels above the control values by 21.6, 9.4, 114, 7.5, and 8.8-folds in serum, cardiac, liver, kidney, and testis samples, respectively, as illustrated in Figure 7. The protection of doxorubicin-treated rats with SML-ANPs succeeded in normalizing MDA levels, as shown in Figure 7, where MDA activity was significantly reduced in serum, cardiac, liver, kidney, and testis by 88.2, 77.3, 80, 72.3, and 81.2%, respectively, in comparison to the corresponding values of the free SML-treated group: 61.9, 55.9, 59, 41.7, and 86.5%, respectively.

### 2.7. Changes in Animal Body Weight and Mortality Rates

As illustrated in Table 3, the body weights (BW) of the experimental animals decreased significantly starting from the first dose in the DOX-treated group compared to the control group (*p* < 0.05). The administration of SML and SML-A NPs controlled this issue. Although there was no significant difference in the animals’ body weight between SML-treated or SML-ANPs-treated animals during the first two weeks, the difference after the third week was highly significant (*p* value < 0.001). Consequently, these findings pointed to the enhanced protective effect of SML-ANPs at the tested dose. Furthermore, during the study period, there was no mortality in both control GP and SML-ANPs GP. However, throughout the experiment, three animals died; two animals in DOX GP and only one animal in SML GP.

**Figure 8:**
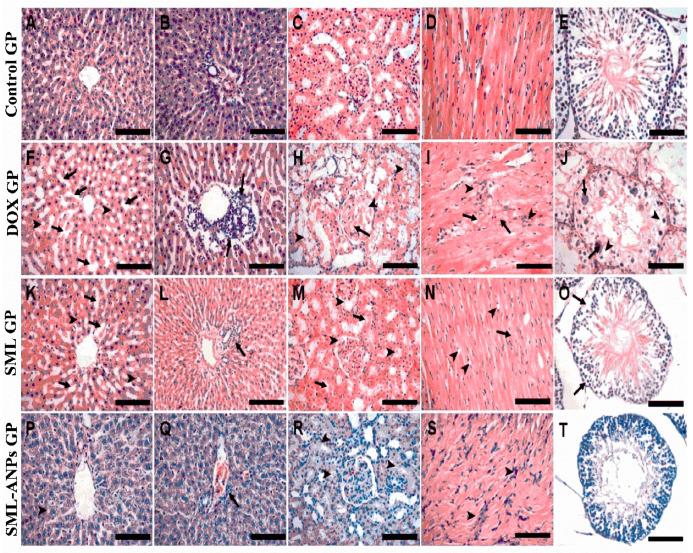
Histopathological changes of heart, liver, kidney, and testes in the control group, doxorubicin treated group (DOX GP), sesamol treated group (SML GP), and sesamol-loaded albumin nanoparticles group (SML-ANPs GP). (**A**–**E**) represent photomicrographs of the liver, kidney, heart and testis sections, respectively, stained by H & E from the control group. (**F**–**J**) represent photomicrographs of the liver, kidney, heart and testis sections, respectively, stained by H & E from DOX GP. (**K**–**O**) represent photomicrographs of the liver, kidney, heart and testis sections, respectively, stained by H & E from SML GP while (**P**–**T**) represent photomicrographs of the liver, kidney, heart and testis sections, respectively, stained by H & E from SML-ANPs GP.

Moreover, the DOX treated group showed remarkable degenerative changes at different grades in the cardiac, liver, kidney, and testis tissues in the form of cellular atrophy, cytoplasmic vacuolization, lymphoid cell aggregation, coagulative necrosis, edema, and hemorrhages. Sesamol counteracted the damaging effects of oxidation in SML GP. However, Sesamol-loaded albumin nanoparticles treatment in SML-ANPs GP markedly ameliorated the DOX-induced pathological changes, and they maintained the normal histological picture, as shown in Figure 8. Moreover, the histopathological lesions in the four groups were scored according to the following: 0 = no damage, 1 = (<25% damage) focal, slight changes, 2 = (25–50% damage) multifocal, significant changes, and 3 = (>50% damage) common widespread changes, as illustrated in the Supplementary material, Table S5.

As shown in Figure 8A,B, the liver from the control group showed normal hepatic cells structure organized in cords (Figure 8A) separated by hepatic sinusoids and radiating from the central vein to the portal area (containing branches of heptaic artery, portal vein, and bile duct) (Figure 8B). Kidney sections from the control group (Figure 8C) showed a normal renal cortex with glomeruli and renal tubules. The heart from the control group (Figure 8D) showed normal cardiac muscle histology with its characteristic striated appearance. Th testicular sections from the control group (Figure 8E) showed normal seminiferous tubule with its normal lining epithelium. The liver sections from the DOX group (Figure 8F,G) showed hepatic cell atrophy: particularly, in the centrilobular area, widening of the hepatic sinusoids (arrows), cytoplasmic vacuolization (arrowheads) (Figure 8F), and focal lymphoid cell aggregations in the portal area (arrows) (Figure 8G). The kidney sections from the DOX group (Figure 8H) showed glomerular necrosis (arrow), accumulation of hyaline casts in the Bowman’s space and the tubular lumen (arrowheads), and severe renal tubular necrosis. DOX group heart sections (Figure 8I) showed severe myocardial necrosis (arrows) and lymphoid cell infiltration (arrowheads). The testicular sections from the DOX group (Figure 8J) showed severe necrosis of the seminiferous tubule with loss of the spermatogenic cells, intratubular multinucleated spermatids (arrows), and cytoplasmic vacuolization (arrowheads). Liver sections from the SML group (Figure 8K,L) showed moderate atrophy of the hepatocytes in the centrilobular zone, moderate widening of the hepatic sinusoids (arrows) (Figure 8K), mild vacuolated cytoplasm (arrowheads) (Figure 8K), and mild lymphoid cell infiltration in the portal area (arrowheads) (Figure 8L). (M) Kidneys from the SML group showed moderate hyaline cast deposition in the Bowman’s space and the tubular lumen (arrowheads) and individual cell necrosis (arrows). The heart sections from the SML group showed mild individual necrosis of the cardiomyocytes (arrow) and moderate cytoplasmic vacuolization (arrowheads) (Figure 8N). The testicular sections from the SML group (Figure 8O) showed almost normal seminiferous tubules and few with depleted spermatogenesis and germ cell detachment from the basal lamina (arrows). The liver sections from the SML-ANPs group (Figure 8P,Q) showed very few hepatocytes with vacuolated cytoplasm (arrowhead) (P) and mild lymphoid cell infiltration in the portal area (arrow) (Q). The SML-ANPs group kidney sections (Figure 8R) showed mild hyaline cast deposition in the tubular lumen (arrowheads). The heart sections from the SML-ANPs group (Figure 8S) showed lymphoid cell infiltration (arrowheads). Finally, the testicular sections from the SML-ANPs group (Figure 8T) showed almost normal seminiferous tubules and spermatogenic cells.

## 3. Discussion

ANPS were prepared by a desolvation technique that did not involve the use of heat and high shear strength, which impair drug stability [29]. The operating conditions such as initial pH of the solution, amount of HSA, and type of desolvating agent have been identified as critical factors that influence characteristics of the formed particles. It was previously reported that the isoelectric point (IP) of HSA is 4.9 [30]. At pH < IP, the absence of electrostatic repulsion forces and hydrophobic interactions led to the aggregation of protein molecules and a larger particle size. Decreasing the overall charges by approaching the IP limits the physical stability of the colloidal dispersion and increases the probability of aggregation. On the other hand, in a basic environment, the electrostatic interactions increased the repulsive forces between HSA molecules and, concomitantly, the enhanced protein–solvent interactions decreased the coagulation. As a result, fine HSA particles could be formed accompanied with a higher loading capacity at pH 8. However, at pH 9, enhanced ionization of the albumin molecules occurred and the high negative charges enhanced the repulsion among the HSA molecules due to steric effects that slowed down the aggregation during particle formation and led to an increase in particle size. Moreover, the molecular repulsion caused by a large magnitude of a negative charge can hinder molecules from getting closer thus preventing the production of aggregates with high polydispersity. Furthermore, the accelerated cross-linking process in alkaline conditions (Schiff’s base formation) could explain the lower loading capacity and the larger particle size. As amino groups are deprotonated at high pH, free amino groups are available for interaction with the aldehydic groups [31].

Figure 2 demonstrates the contour plots of the D-optimal models. Noticeably, from the plots, a lower concentration of the HSA solution during desolvation significantly decreased (*p* < 0.0001) the particle diameter and increased the loading capacity. Furthermore, increasing the amount of HSA affected the coagulation of the albumin molecules and the formation rate of ANPS. The free albumin molecules start to condense around the protein nuclei, forming albumin particles that start to aggregate with a larger particle size and a high percentage of yield. Upon increasing HSA concentration, the viscosity of the solution increases. As a result, the movements of HSA molecules become slower between the aqueous phase and the desolvating agent. This contributed to a slower nucleation rate and hence larger particles. Changing the desolvation agent from a polar ethanol to an ethanol acetone mixture significantly affected the ANPs zeta potential. Albumin molecules are insoluble in both ethanol and the mixtures of ethanol and acetone [32]. Consequently, this promotes albumin molecules nucleation and then aggregation. The aggregation process results spontaneously in ANPs formation. Notably, the higher hydrophobicity of acetone resulted in particles that possess a lower charge, compared to particles formed with using pure ethanol. The high negative charge of NPs could improve their stability. Efficacy of nanoparticles after injection is mainly dependent on particle size and surface charge. The D-Optimal design allowed analysis of the factors that influence characteristics of the formed nanoparticles.

The experimental results confirmed the ability of using sucrose and trehalose as cryoprotectants to prevent the aggregation of ANPs during the freeze-drying process and to protect them from the deterioration effect of dehydration and the subsequent re-hydration step [33]. Mannitol failed to prevent the particles’ aggregation. This could be ascribed to its partial crystallization, mostly during the freeze-drying process. The ability of sucrose and trehalose to form amorphous glasses that are capable of interacting with amorphous proteins, forming a glass matrix around nanoparticles during freeze-drying, could interpret their effectiveness over mannitol [34].

In the in vitro release profile of SML-ANPS, the initial fast release can be attributed to the desorption and the diffusion of SML from the outer surface of ANPS. The initial drug release is necessary for a satisfactory therapeutic effect. The slow release is mainly because of the slow diffusion of SML through the albumin matrix, and this was inconsistent with similar data documented by other authors [35]. On the other side, glutaraldehyde would disperse inside the nanoparticle network throughout the cross-linking process and associate covalently with adjacent amine groups of lysine in albumin residues, leading to a strong stabilization of the matrix of ANPS, resulting in a slow release of SML. During circulation in the blood, slower drug release from the nanoparticles is beneficial as this minimizes the systemic adverse side effects, and it increases ANPs’ targeting ability.

SML has been previously tested on different cell lines as an antioxidant to protect them against the damaging effect of cancer therapy. [36]. Sesamol could reduce the oxidative damage and the toxicity to H9c2 cardiomyoblasts caused by doxorubicin [37]. The cellular ability to reduce MTT dye is an indicator of mitochondrial activity, a measure of cellular viability [38]. DOX triggers cellular apoptosis by increasing the levels of intracellular ROS, thus a reduction in cell viability and increasing apoptosis were observed to >70% [39]. However, co-treatment with an antioxidant agent counteracts ROS production and improves the endogenous enzyme levels. Sesamol, being a phenolic compound possessing a methylenedioxy group, is considered a potent inhibitor of cytokine production. Moreover, it is a powerful antioxidant [40]. Furthermore, sesamol was previously reported to elevate mRNA levels and the protein expression of the antioxidant enzymes HO-1 and NQO1 as well as to decrease the inflammatory cytokines TNF-α and IL-1β in D-galactose-treated mice serum. Moreover, the activity of CAT and the GSH level were found to increase in sesamol-treated mice serum. Additionally, sesamol treatment was also found to balance the cellular redox status, protect against mitochondrial dysfunction, and upregulate the antioxidant enzymes by activating the Nrf2 transcriptional pathway [41].

Therefore, treatment with both free and encapsulated sesamol had a protecting effect. However, higher hepato-protectivity of the used antioxidant drug was observed when it was encapsulated in albumin nanoparticles compared to the free drugs. This can be explained by the efficient internalization, localization, and endocytic uptake of albumin nanoparticles via different pathways [42]. Particles with diameters < 200 nm could be phagocytized by cancer cells due to the enhanced permeability and a retention effect [43]. A synergistic antioxidant effect may be also ascribed to the presence of albumin. HSA was reported to possess direct and indirect antioxidant effects. It exerts its direct effect being a free radical scavenger where the free thiol groups in the Cys-34 residue are usually able to trap different types of ROS and due to the presence of six methionine groups that are easily oxidized. HSA can also act indirectly by binding bilirubin that inhibit lipid peroxidation and free metals such as iron and copper, which catalyze aggressive ROS formation [22].

Furthermore, anionic HSA NPs bind to the albumin receptors on the cell surface e.g., Gp60, SPARC. Additionally, binding of albumin to the neonatal Fc receptor (FcRn), a cell membrane bound receptor found on endothelial cells and various organs, such as the kidneys, liver, and intestine, was elucidated by Anderson and co-workers [42]. As SML water solubility impaired its cellular uptake and such molecules need a specific receptor or transporter to transfer it into the cells, it could therefore be hypothesized that ANP improved the availability of SML by prolonging drug residence time and increasing tissue uptake. Antioxidative agents inhibit oxidative damage and death in normal cells by the induction of endogenous antioxidants and/or reactive oxygen species scavenging. Moreover, they prevent the activation of chemical resistance pathways in cancer cells through oxidative stress [44].

SML-ANPs and SML solution pharmacokinetic profiles were studied. SML solution was directly injected, and thus blood exposure resulted in faster elimination. However, the ANPs acted as reservoirs for SML in the blood, where SML-ANP was gradually degraded over time and SML was slowly released into the bloodstream, resulting in an extended residence time, a slower clearance, and higher AUC.

Anticancer agents such as DOX have been demonstrated to exert serious dose impacts on the other non-targeted tissues. Previous reviews corelated DOX’s toxic side effects with several factors, such as ROS and RNS generation [25,26]. The semiquinone formed by DOX reacts with molecular oxygen, providing a radical superoxide (O2^−^) and returns to its quinone form. This cycling process produces several active oxidant species, H_2_O_2_, OH^−^, and ONOO^−^. The elevated levels of free radicals have a significant potential to initiate lipid peroxidation upon rapid interaction with lipids. Furthermore, it triggers an oxidative stress that affects different biomolecules such as the membrane-bound proteins, enzymes, lipids, mitochondrial genomes, and others. Several studies have shown that oxidative stress and development of iron anthracycline free radicals may be highly responsible for the pathogenesis of DOX-induced cardiotoxicity, hepatotoxicity, nephrotoxicity, and testicular toxicity [45]. DOX-induced cardiotoxicity in rat models is manifested as an escalating level of serum lactate dehydrogenase (LDH), creatine kinase, and cardiac malondialdehyde (MDA), which are associated with the histopathological changes. Coincidentally, DOX has a high affinity for cardiolipin, a phospholipid inserted in the cell membrane of mitochondrial where the DOX-cardiolipin complex act as a substrate and start the lipid peroxidation process. When the hepatocytes are damaged, certain changes occur in cell membrane permeability, which is further correlated with the release of enzymes from the cells, reducing the levels of ALT and AST and increasing their serum levels. A previous study reported an enhanced lipid peroxidation in the kidneys of DOX-treated rats. A recent study provided both biochemical and histological evidence for marked reproductive toxicity caused by DOX through oxidative stress. It was reported that DOX-treated rats showed considerably higher levels of MDA suggesting the strong pro-oxidative activity of DOX. The formation of free radicals is considered to be the rate limiting step in lipid peroxidation. Compared to the DOX-treated group, the administration of SML-ANPS 30 min before DOX showed substantial improvement in the cardiac markers (CK and LDH serum levels) and the biochemical variables. Normally, during DOX therapy, extensive free radical production results in increased membrane permeability of MDA in tissue homogenates as MDA molecules are normally located in the cellular cytoplasm and leak into the serum after tissue damage during inflammation, which can explain histological injuries. Similarly, other studies showed an increase in the levels of biochemical markers with an elevation of LPO in different organs after doxorubicin treatment [11]. It was reported that the therapeutic indices in traditional chemotherapy were enhanced upon co-treatment with antioxidants [44].

## 4. Materials and Methods

### 4.1. Materials

The Bradford reagent (Cairo, Egypt), Doxorubicin, was purchased from Thermo Fisher Scientific (Fair Lawn, NJ, USA); Glutaraldehyde, 25 wt.% solution was purchased from Acros Organics (Merelbeke, Belgium); Fetal Bovine serum, gentamycin, HEPES buffer, L-glutamine and RPMI-1640 medium were purchased from Lonza (Verviers, Belgium); HPLC grade acetone, tetrazolium salt (MTT), sesamol and trypan blue dye were purchased Sigma-Aldrich (St. Louis, MO, USA); HPLC grade acetonitrile was purchased from Carl Roth (Karlsruhe, Germany); HPLC grade ethanol was purchased from Thermo Fisher Scientific (Schwerte, Germany); lyophilized HSA were purchased from Alfa Aesar (Kandel, Germany); and the Milli-Q^®^ Integral Water Purification System for Ultrapure Water was purchased from Merck Millipore (Darmstadt, Germany).

### 4.2. Methods

#### 4.2.1. Fabrication of SML-ANPs

ANPs were prepared using the desolvation process. Briefly, different concentrations of HSA solution were prepared (25, 50 and 100 mg/mL). Then, the pH of drug-polymer solution was adjusted at different pH values (7, 8, and 9) using a phosphate buffered saline solution (PBS). SML was dissolved in the HSA solution and incubated for 4 h under constant stirring. Using a burette, a desolvating agent (ethanol or ethanol/acetone, 1:1) was added at a constant rate of 1 mL/min under stirring of 550 rpm, until turbidity just appeared. Finally, the formed ANPs were cross-linked by 8% glutaraldehyde (GA) overnight at room temperature. The unbound drug in the nanoparticle dispersion was removed by three cycles of washing and centrifugation at 14,000 rpm for 30 min each using a cooling centrifuge at 4 °C (Sigma Laboratory centrifuges, Osterode am Harz, Germany). The supernatant was collected for further analysis. After each cycle, 5 min sonication was used for the re-dispersion of the formulation.

#### 4.2.2. Characterizations of Albumin Nanoparticles

The average particle size (P.S.) and polydispersity (PDI) of purified reconstituted nanoparticles were measured using dynamic light scattering (DLS) and utilizing a Malvern Zetasizer (Worcestershire, UK). The samples were diluted at a ratio of 1:10, with purified water. Thereafter, they were measured using the backscattered light detector operating at angle of 173° and the Zeta potential values were measured by laser Doppler anemometry. All samples were measured at 25 °C in triplicate to assess the reproducibility. Quantification of unbound SML in the supernatant was performed using a Dionex Ultimat 3000 UHPLC system (Thermo Scientific, Waltham, MA, USA) equipped with HPG-3200 RS pump, a DAD-3000 RS detector, a WPS-3000TRS analytical autosampler, and a Hypersil BDS C-18 column with the dimensions of (5 µm, 4.6 × 150 mm). A reversed-phase technique was proceeded using a mobile phase consisted of acetonitrile and 0.3 M KH2PO4, pH 3.5, in the volume ratio 70:30, respectively. The mobile phase flow rate was 1 mL/min, the injection volume was 20 μL, and the eluent was quantified by Diode-Array Detection (DAD) detector at 294 nm wavelength. The method accuracy was determined to be 99.99% with a limit of quantification (LoQ) of 100 ng/mL. Then, encapsulation efficiency (% EE) was calculated according to Equation (1);

(1) $$\%\;\mathrm{EE}=\frac{\mathrm{Initial}\;\mathrm{amount}\;\mathrm{of}\;\mathrm{SML}-\mathrm{amount}\;\mathrm{of}\;\mathrm{unloaded}\;\mathrm{SML}\;}{\mathrm{Amount}\;\mathrm{of}\;\mathrm{drug}\;\mathrm{initially}\;\mathrm{added}\;}\times 100,$$

while to determine the percent of ANPs yield, the unbounded HSA concentration in the supernatant was determined spectrophotometrically at 595 nm, using a pre-constructed calibration curve (10–80 µg/mL), based on the method of Bradford [46]. The percentage of drug loading capacity (% LC) and the yield (% Y) of the nanoparticles were calculated according to Equations (2) and (3), respectively;

(2) $$\%\;\mathrm{Yield}=\frac{\mathrm{Initial}\;\mathrm{amount}\;\mathrm{of}\;\mathrm{HSA}\;-\;\mathrm{amount}\;\mathrm{of}\;\mathrm{unbounded}\;\mathrm{HSA}\;}{\mathrm{Initial}\;\mathrm{amount}\;\mathrm{of}\;\mathrm{HSA}}\times 100$$

(3) $$\%\;\mathrm{LC}=\frac{\mathrm{Amount}\;\mathrm{of}\;\mathrm{encapsulated}\;\mathrm{SML}\;}{\mathrm{Total}\;\mathrm{weight}\;\mathrm{of}\;\mathrm{SML}-\mathrm{ANPs}}\times 100.$$

For the interpretation of Fourier transform-infrared spectroscopy (FT-IR) and differential scanning calorimetry (DSC) thermograms of lyophilized SML, HAS, SML/HSA physical mixture, SML/HSA mixture after incubation, and SML-ANPs see supplementary material, Figures S1 and S2.

#### 4.2.3. Design of Experiments (DOE) for Studying Critical Factors

Three levels for each of the investigated factors were chosen to generate an experimental design, namely, the D-optimal design (DOD) (Table 4). The concentration of HSA, pH of albumin-drug solution, and the type of the desolvating agent were treated as three independent variables affecting the SML-ANPs formulation. The design matrices comprised 18 experimental runs (see supplementary material, Table S1).

#### 4.2.4. In Vitro Drug Release of SML from Selected SML-ANPs

An optimal amount of selected SML-ANPs containing 5 mg of SML were suspended in phosphate buffered saline at pH 7.4 in a dialysis membrane (Molecular weight cut-off 12 kDa) and dialyzed against 50 mL of phosphate buffered saline. The medium was continuously stirred at 100 rpm and maintained at a temperature of 37 °C. Samples were withdrawn at predetermined time intervals and the same volume was replaced with prewarmed fresh medium. The SML quantification was preformed, using HPLC as previously described in the encapsulation determination section.

##### Release Kinetic Studies

To study the release kinetics of SML, the data obtained from the in vitro release studies of selected SML-ANP were fitted to various kinetic models, such as zero-order, first-order, and the Higuchi, Korsmeyer–Peppas, and Hixson Crowell models [47]. Regression analysis was used to determine the constants and the corr4elation coefficients of the data (R^2^) [48].

#### 4.2.5. Morphological Characterizations

The morphology of selected SML-ANPs was visualized by Quanta FEG 250 scanning electron microscopes (SEM) at a magnification of 120,000× and an accelerated voltage of 20.0 kV. The sample was prepared by pipetting 10 μL of the SML-NPs suspension (2 mg/mL) onto copper tape and leaving it to dry.

#### 4.2.6. Freeze Drying of the Selected SML-ANPs

Using CHRIST Alpha 2-4 LD plus freeze-dryer (Osterode am Harz, Germany), the ability of the selected SML-ANP to maintain their physiochemical characterizations under freeze drying conditions was studied in absence and in the presence of distinct types of cryoprotectants as trehalose, mannitol, and sucrose. The cryoprotectants were evaluated at three different concentrations of 1%, 2%, and 3%, in order to obtain a stable solid dosage form without negative impact on the product properties. In triplicate, samples and the respective control without cryoprotectants were frozen at −80 °C overnight prior to freeze drying process. The vacuum pressure was set at 0.011 mbar and the temperature was maintained at −60 °C for 48 h. After freeze drying, the samples were re-dispersed in distilled water. The particle size and the PDI were measured and compared with their respective characteristics before freeze-drying [49].

#### 4.2.7. Evaluating the Reconstituted SML-ANPs Stability

To evaluate the stability of SML-ANPs, three batches of the selected formulation after freeze drying were stored in sealed Eppendorf tubes (1.5 mL) at 4 °C for 6 months and then reconstituted for analysis. The reconstituted freeze dried SML-ANP formulations were evaluated in terms of particle size and the polydispersity index. Besides, further experiments were performed to examine the stability in different biological media after a 2 h incubation. The nanoparticles were diluted 1 in 20 with purified water, phosphate buffered saline (PBS), and Dulbecco’s Modified Eagle Medium (DMEM), and three readings were taken at after 2 h.

#### 4.2.8. The Hepatoprotective Study of SML-ANPs

Sprague–Dawley male rats (200–250 g) were obtained from the Animals House in the Faculty of Science at Al-Azhar University, Cairo, Egypt and treated according to Helsinki’s declaration of animals use (2008). Before performing liver biopsies on five rats, they were administered ether anesthesia. The hepatocyte isolation was performed according to the collagenase perfusion procedure which was previously described by Reese and Byard [50]. The hepatocytes (1 × 10^6^ cells/mL) were placed in a Krebs–Henseleit buffer (pH 7.4) containing 12.5 mM HEPES and kept at 37 °C with 95% O_2_ and 5% CO_2_. The hepatocytes with a viability of more than 90%, which was measured with Trypan Blue, were used in the experiments [51]. Rat hepatocytes were exposed to a medium containing DOX (1µM). Then, the viability of cells was estimated by the MTT reduction assay [52]. The experimental groups tested were as follows: Control group: untreated rat hepatocytes; DOX-treated group: rat hepatocytes were treated with DOX (1 µM); SML treated group: rat hepatocytes were treated with DOX (1µM) and a nontoxic dose of free SML; and SML-ANPs treated group: rat hepatocytes were treated with DOX (1 µM) and a nontoxic dose of SML-ANPs. In case of tested formulations treatment, the hepatocytes were pretreated with either SML or SML-ANPs 24 h before adding the DOX. Each treatment was repeated four times (i.e., four wells for each treatment). The doses of DOX and SML were selected according to a preliminary cytotoxicity study and previous literature (supplementary file, Figure S3 and Table S6) [53,54].

#### 4.2.9. Pharmacokinetic Study

Adult male healthy Wister albino rats (weighing in the range of 150 ± 20 g) were obtained from the animal house in the Faculty of Medicine, Assiut University, Assiut, Egypt. The rats were acclimatized for 7 days and maintained on a 12:12 h light/dark cycle, and they had free access to food and water. The protocol of the study (no. 155) was approved by the animal care and use committee of the Faculty of Pharmacy, Ain Shams University, Cairo, Egypt. A pharmacokinetic study was performed to assess SML sustained release from SML-ANPs and to estimate their ability as a drug carrier to modulate SML rapid clearance. Eight rats were randomly divided into two groups (four animals per group). The first group received a single dose of free SML solution (15 mg/kg), while the second received an SML-ANPs dispersion loaded with an equivalent amount to the injected SML solution. Blood samples were withdrawn via vein puncture from the caudal vein into heparinized tubes at predetermined time intervals (0.25, 0.5, 2, 4, 8, 12, and 24 h) after intravenous injection (IV). Then, plasma samples were separated by centrifugation at 3000 rpm for 10 min and stored at −20 °C for further analysis. Plasma SML concentrations were analyzed using a reversed phase-HPLC by the method described previously with some modifications [55,56]. The mobile phase composed of 70:30 *v/v* acetonitrile to 0.3 M orthophosphate buffer adjusted at pH 3.5 by phosphoric acid. The flow rate was set at 1.0 mL/min, the injection volume was 20 μL, and elute was analyzed with a DAD detector set at a wavelength of 294 nm. Acetonitrile (1 mL) was added to the plasma sample (200 μL) to precipitate the plasma proteins [57]. Then, plasma samples were vortexed for 10 min and then incubated and centrifuged at 4000 rpm for 15 min. The collected supernatants were filtered through a 0.45 μm syringe filter (Millipore, Billerica, MA, USA). Then, they were analyzed using the HPLC against a calibration curve of SML in the plasma that was constructed using blank plasma spiked and mixed with standard SML solutions to obtain a concentration range of 0.01–10 μg/mL. The spiked plasma samples were then subjected to the same extraction procedure as the tested plasma samples to avoid any quantitation error factor in sample preparation [55,57]. The pharmacokinetic parameters were calculated by fitting the plasma concentration–time data to a suitable model using WinNonlin Professional Edition software version 2.0 (Science Consulting, Apex, NC, USA). The maximum plasma concentration (C_max_), the time of maximal concentration (T_max_), the half-life (T_1/2_), the area under the curve (AUC0–∞), and mean residence time (MRT0–∞) were calculated.

#### 4.2.10. *In Vivo* Study of SML-ANPs

Acute administration of DOX in Wister rats, which normally induces a state of oxidative stress, is considered a widely used animal model to assess the protective ability of antioxidative agents. An optimal dose was chosen according to a preliminary study and following previous literature to be sufficient to induce a progressive oxidative state in rats. This study was carried out in adult male healthy Wister rats weighing in the range of 160 ± 30 g from the Animal Facility of the Faculty of Medicine, Assiut University, Egypt. The protocol of the study was approved by the Institutional Animals Ethics Committee of the College of Pharmacy, Ain Shams University (No. 155). Rats were acclimatized for 7–10 days before the initiation of the experiment to observe any sign of disease, they were maintained on a 25 ± 5 °C 12/12 h light/dark cycle, and they had free access to food and water.

Rats were randomized into four groups, each group containing 10 animals: the control group received saline four times per week, during three weeks (control group); the DOX group received a repeated dose of DOX (2.5 mg/kg, intraperitoneal injection (IP)) three times per week during the first two weeks and received just saline during the third week; the SML group received an SML solution 30 min before the administration of DOX during the first two weeks and before saline during the third week; and the SML-ANPs group received SML-ANPs 30 min before the administration of DOX during the first two weeks and saline during the third week. The body weights and the mortality rate of animals were recorded during the experiment. At the end of the study, the blood samples were collected and the animals were sacrificed to take samples from different organs for further examination, (see supplementary file).

##### Biochemical Assessment of the Selected SML-ANPs Formulations

Creatine kinase (CK) and lactate dehydrogenase (LDH) serum levels were detected as markers of cardiotoxicity with respective kits and with the help of an ELISA reader (Bio Tek; Santa Clara, USA) according to the manufacturer’s protocols. Nevertheless, the liver injury was evaluated by measuring serum alanine aminotransferase (ALT) and aspartate transaminase (AST) levels as a specific biomarker using a specific kit following the manufacturer’s protocol. Furthermore, the biochemical determination of malondialdehyde (MDA) serves to indicate lipid peroxide formation. MDA levels (marker of lipid peroxidation (LPO)) were assessed by reaction with thiobarbituric acid (TBA) at 100 °C. The reaction between MDA and TBA produces a pink pigment, which has a maximum absorption at 532 nm. Briefly, 50 µL of supernatant, 1 mL of TBA, and 1 mL of trichloroacetic acid (TCA) [0.75% TBA: 30% TCA] were mixed, placed in a boiling water bath for 60 min, and cooled and centrifuged for 15 min at 4000 rpm. The supernatant absorbance was measured against a reference blank at 532 nm by spectrophotometer; 1,1,3,3-Tetramethoxypropane (Sigma Chemicals, St. Louis, MO, USA) was used as a standard MDA. The results were expressed as n mol/mg protein.

##### Histopathological Analysis

Since the histological study of doxorubicin multi-organ toxicity is a wide topic, we focused on the toxicity of vital organs and a reproductive system that can be harmed by cancer and its associated treatments, and we evaluated the protective activity of our formulation against organ toxicity reported in previous literature [45,58,59,60,61,62]. Liver, kidney, heart, and testis specimens were cut and fixed in a 10% neutral buffered formalin. The formalin-fixed samples were routinely processed, embedded in paraffin, and sectioned. Serial 3 μm sections, serially dehydrated in an ethanol gradient, were stained with Mayer’s hematoxylin (Merck, Darmstadt, Germany) and eosin (Sigma, St. Louis, MO, USA) and examined microscopically. The histological evaluation was performed by a histopathologist (Dr Mahmoud Soliman) in a blind fashion on coded samples, and a comparison was made with the sections from the control.

### 4.3. Statistical Analysis

All experiments were performed in triplicate and results were expressed as means ± standard deviations (SD) except for the in vivo study where the results were expressed as mean ± standard error (SE) for eight animals in each group (n = 8). Statistical analysis tests were performed using SPSS 21.0 software. For all results, the differences were considered significant when *p* < 0.05, and they were regarded as extremely significant when *p* < 0.0001 presented by * and ***, respectively. Comparisons between groups were performed using one-way multivariate ANOVA followed by a Tukey’s post hoc test or Student t-test where appropriate.

## 5. Conclusions

Based on the findings presented in this study, it is possible to conclude that the desolvation method successfully produced SML-ANPs with promising physicochemical features, such as high stability, sustained release and high loading capacity, where the particle size, zeta potential, and loading capacity of the selected formulation were 127.24 nm, −26.21 mV, and 96.89 g/mg ANPs, respectively. In addition to the specific role of albumin molecules in cellular endocytosis, particles with diameters less than 200 nm improve cellular absorption. Albumin nanoparticles also enhanced pharmacokinetic characteristics. Sesamol suffer from low bioavailability, and they displayed a short half-life and a rapid elimination rate of 1.25 h and 0.53 L/h, respectively. However, albumin can act as an SML reservoir in the bloodstream with an extended half-life and slower elimination rates of 8.9 h and 0.11 L/h, respectively. SML-ANPS exhibited significant protective effects against hepatocytes pretreated with doxorubicin and the DOX-induced acute toxicity in albino rats as an oxidative stress animal model by down regulating the production of harmful free radicals and the LPO signaling pathway in serum and different organ tissues. Co-treatment with SML-ANPs significantly reduced the elevated serum CK and LDH by 59.3 and 64.5%, respectively, while AST and ALT were inhibited by 60.5 and 59% in comparison to the corresponding values of the free SML treated group: 35.6, 47.2, 49.9, and 41.2%, respectively. As a result, sesamol-loaded albumin nanoparticles may be considered a viable and potentially clinically applicable nano-based platform for the treatment of cancer and inflammatory illnesses in the future. However, more research into their medicinal potential is necessary before use in clinical application.

## Figures and Tables

**Table 1:** Characteristics of the generated response surface models.

Responses	PS	PDI	Zeta	%EE	%Yield	LC ^1^
Order	Quadratic	Quadratic	Linear	Quadratic	Quadratic	Quadratic
Significance	extremely significant (*p* < 0.001)	ns	extremely significant (*p* < 0.001)	extremely significant (*p* < 0.001)	extremely significant (*p* < 0.001)	extremely significant (*p* < 0.001)
R^2^	0.994	0.649	0.969	0.972	0.983	0.942
Adjusted R^2^	0.989	0.338	0.963	0.947	0.968	0.890
Predicted R^2^	0.969	−0.468	0.953	0.871	0.923	0.736
Adequate precision	45.801	3.998	34.93	18.01	23.926	12.855 ^2^

^1^ Abbreviations: DL: Drug loading; EE%: Percent of entrapment efficiency; SML-ANPS: Sesamol-loaded albumin nanoparticles; PDI: Polydispersity index; PS: Particle size; Y%: Percent of yield; and ZP: Zeta potential. ^2^ “ns” presents that no significant differences.

**Table 2:** Pharmacokinetic parameters of SML concentration after intravenous administration of free SML and SML-ANPs to rats.

Parameters	t1/2(h) *	AUC (µg/mL h) *	MRT(h) *	CL (mL/h) *
Free SML	1.3 ± 0.13	19.5 ± 4.2	2.6 ± 0.03	528.2 ± 115.5
SML-ANPs	6.1 ± 0.44	88.7 ± 12.2	8.5 ± 0.63	114.2 ± 15.9

Abbreviations: t½, half-life; AUC, area under the curve; MRT, mean residence time; CL, clearance, SML: free sesamol and SML-ANPS: Sesamol-loaded albumin nanoparticles. Notes: For all results, the differences between SML-ANPs vs. free SML were considered significant when *p* < 0.05 presented by *. All results expressed mean ± SD, n in each group = 4.

**Table 3:** Body weights of the experimental animals after the administration of the investigated formulations as compared to the control.

Wt.(g)/Survival Rate	Control GP	DOX GP *	SML GP ^ns^	SML-ANPs GP ^ns,1,2^
Body weight	151.7 ± 11.02	101.26 ± 5.61	146.5 ± 14.12	162.44 ± 15.49
Survival rate	10/10	8/10	9/10	10/10

^1^ Abbreviations: DOX GP: Doxorubicin treated group; SML GP: Sesamol treated group; SML-ANPs GP: Sesamol loaded albumin nanoparticles treated group. ^2^ For all results, compared to the control group ^ns^ presents that no significant difference was found and the differences were considered significant when *p* < 0.05 presented by *. Body weight results expressed mean ± SD (n in each group = 10).

**Table 4:** Factors controlling SML-ANPs preparation and their levels in D-optimal design.

Independent Variable	Level (−1)	Level (0)	Level (+1)
PH	7	8	9
HSA (mg/mL)	25	50	100
D.A	Ethanol	-	Ethanol/acetone

Abbreviations: D.A: Desolvating agent; HSA: Humane serum albumin; SML-ANPS: Sesamol-loaded albumin nanoparticles.

## Data Availability

Data is contained within the article and supplementary materials.

## Supplementary Materials

The following supporting information can be downloaded at: https://www.mdpi.com/article/10.3390/ph15060733/s1, Figure S1: DSC thermograms of lyophilized SML, HAS, SML/HSA physical mixture, SML/HSA mixture after incubation, and SML-ANPs; Figure S2: FTIR spectra of HSA (A) SML (B) SML/HSA physical mixture (C) SML/HSA mixture after incubation (D), and SML-ANPS (E) together with the successful docking of sesamol on HSA (F); Table S1: Physicochemical characteristics of SML-ANPs; Table S2: The effect of the factors controlling SML-ANPs preparation and their levels in the D-optimal design, where pH is the most significant factor; Table S3: Release kinetics of SML-ANPs (F14); Table S4: Hepatoprotective effect of free SML and SML-ANPs on rat hepatocytes measured by the MTS assay; Table S5: The histopathological scores of the liver, kidney, heart, and testis in each groups ^a^; Figure S3: Dose-response curve for sesamol effects on cell viability of rat hepatocytes to calculate the CC 50. The values are presented as mean ± SD (n in each group = 3); Table S6: Evaluation of sesamol cytotoxicity against Rat hepatocytes cell line after 48 h [50,63,64,65].

## Abbreviations

ALT: Serum alanine aminotransferase; ANOVA: Analysis of variance; ANPs: albumin-based nanoparticles; AST: Aspartate transaminase; AUC0–∞: The area under the curve; CK: Creatine kinase; C_max_: The maximum plasma concentration; CV%: Coefficient of variation; DAD: Diode-Array Detector; DL%: Percent of the drug loading; DOE: Design of experiments; DOD: D-optimal design; DOX: Doxorubicin; DLS: dynamic light scattering; DSC: Differential scanning calorimetry; EE%: Entrapment efficiency; Fe^2+^: iron; FT-IR: Fourier transform-infrared spectroscopy; GA: glutaraldehyde; GP: Group; HO•: hydroxyl radical; HSA: Human serum albumin; IV: intravenous injection; LDH: Lactate dehydrogenase; LoQ: Limit of quantification; LPO: Lipid peroxidation; MDA: Malondialdehyde; MRT0–∞: Mean residence time; MTT: 3-(4,5-Dimethyl-2-thiazolyl)-2,5-diphenyl-2H-tetrazolium bromide; USA: The United States of America; PBS: Phosphate buffered saline solution; PDI: Polydispersity index; PS: Particle size; ROS; reactive oxygen species; SD: Standard deviations; SE: Standard error; SEM: Scanning electron microscopes; SML: sesamol; SML-ANPS: Sesamol-loaded albumin nanoparticles; SPSS: Statistical Package for the Social Sciences; TBA: Thiobarbituric acid; TCA: Trichloroacetic acid; T1/2: the half-life; T_max_: the time of maximal concentration; %Y: Yield; ZP: Zeta potential.
