# Supplementary material for: Sesamol Loaded Albumin Nanoparticles: A Boosted Protective Property in Animal Models of Oxidative Stress
Source: Pharmaceuticals (Basel). 2022 Jun 10;15(6):733. doi: 10.3390/ph15060733 (PMC9228363; doi:10.3390/ph15060733)
Supplement: Supplementary file 1 [file pharmaceuticals-15-00733-s001.zip › pharmaceuticals-1697247-supplementary.pdf]

Supplementary Data

The generated equations according to the DOD were as follows (Eqs. 9-14):

$$\text{Size} = 148.84 + 30.63 * \text{A} - 44.92 * \text{B} - 8.28 * \text{C} - 9.17 * \text{AB} - 1.31 * \text{AC} + 2.59 * \text{BC} - 8.81 * A^2 + 118.81 * B^2 \quad \text{Eq. 9}$$

$$\text{PDI} = 0.1295 - 0.0177 * \text{A} - 0.0182 * \text{B} - 0.0215 * \text{C} + 0.0029 * \text{AB} + 0.0209 * \text{AC} - 0.0218 * \text{BC} + 0.0003 * A^2 + 0.0568 * B^2 \quad Eq. 10$$

$$\text{Zeta} = -29.94 - 1.28 * \text{A} - 10.03 * \text{B} + 3.30 * \text{C} \quad Eq. 11$$

$$\%\text{EE} = 75.10 + 5.95 * \text{A} - 16.74 * \text{B} + 6.73 * \text{C} - 1.13 * \text{AB} - 0.8168 * \text{AC} - 4.67 * \text{BC} - 1.57 * A^2 - 33.46 * B^2 \quad Eq. 12$$

$$\%\text{Y} = 82.63 + 18.38 * \text{A} - 1.77 * \text{B} + 5.61 * \text{C} - 2.17 * \text{AB} - 0.0882 * \text{AC} + 0.9092 * \text{BC} - 12.39 * A^2 - 2.09 * B^2 \quad Eq. 13$$

$$\%\text{LC} = 79.22 - 24.62 * \text{A} - 17.09 * \text{B} + 2.90 * \text{C} + 8.66 * \text{AB} - 0.8412 * \text{AC} - 4.46 * \text{BC} + 1.31 * A^2 - 36.73 * B^2 \quad Eq. 14$$

Differential scanning calorimetry

DSC analysis was performed to detect any change in the physical state of SML in the nanoparticles. DSC thermograms of pure SML, HSA, physical mixture of SML/HAS, mixture of SML/HAS solution lyophilized after 24-h incubation and SML-ANPs, were recorded. As shown, SML thermogram shows two peaks. A sharp typical melting endothermal peak of the drug was recorded at 65.3 ºC and the degradation peak starts to appears around 180 ºC, as previously reported [1]. HSA demonstrated two broad endothermic peaks at 53.5 and 209 ºC. The physical mixture thermogram indicated that HSA did not change the physical state of SML [2]. SML/HSA incubated mixture showed a change and broadening in the melting peak of sesamol, thus indicating an interaction between albumin and sesamol. Moreover, SML-ANPs

thermogram did not show the characteristic endothermic peaks of SML at 65.3 °C indicating SML crystallization was inhibited by albumin during nanoparticles formation, thus the drug is no longer existing in the crystalline form [2].

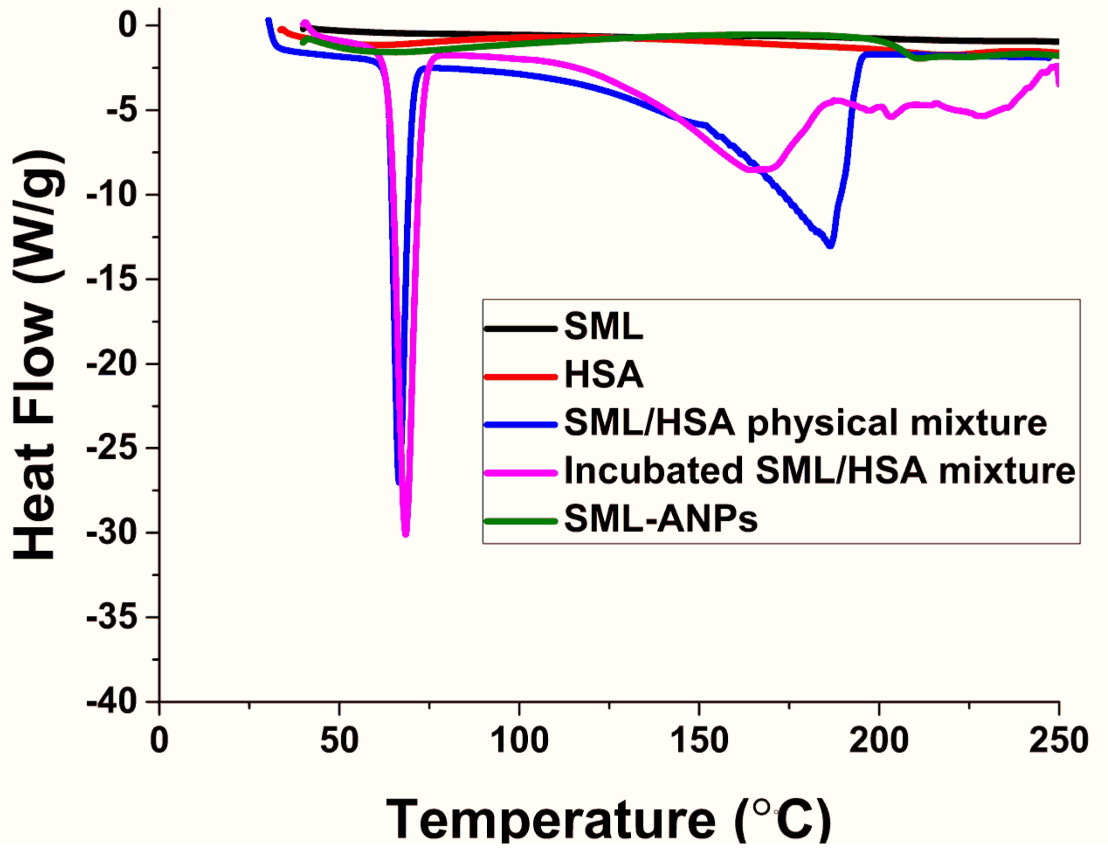

Figure S1. DSC thermograms of lyophilized SML, HAS, SML/HSA physical mixture, SML/HSA mixture after incubation, and SML-ANPs.

Fourier transform-infrared spectroscopy (FTIR analysis)

FTIR analysis between 2000 and 600 cm-1 were recorded and presented to illustrate the conformational changes of SML, HSA, SML/HAS mixture, SML/HAS 4-h incubated mixture and SML-ANPs. The FTIR spectra of lyophilized HAS showed two characteristic groups; the amide and hydroxyl group. The amide group showed two bands around 1539.54 and 1632 cm^{-1} correspond to C-N stretching coupled with N-H bending and the C-O stretching frequency, respectively. A typical broad hydroxyl group band could be identified at 3309.44 cm^{-1}. For sesamol, the characteristic broad band of -OH group appeared at 3213.1 cm^{-1}, two bands at 1035.96 and 843.38 cm^{-1} represent the C-O stretching vibrations peaks. The

symmetric and asymmetric C-O-C stretch band of the methoxy group could be identified at 1039.28 and 1269.59 cm^{-1}, respectively. The two peaks of 1471.98 & 1502.14 cm^{-1} represents the C=C group of the aromatic ring. In SML/HAS physical mixture, all characteristic peaks of sesamol appeared without remarkable changes *e.g.* -OH and CO groups. In the spectrum of incubated SES/HAS mixture and SML-ANPs, the band at 843.38 cm^{-1} which represent CO of the hydroxyl group almost disappeared from the spectrum and the intensity of the band at 1039.28 & 1269.59 cm^{-1} of C-O-C remarkably decreased. The disappearance of C-OH peak, supports the assumption that sesamol interacts with albumin by hydrogen bonding (Min et al., 2004). Furthermore, previous spectroscopic studies supported the hypothesis that the hydroxyl group in phenolic compounds such as sesamol can interact with the C=O and C–N groups in protein polypeptide chains besides the aromatic hydrophobic interaction and had a significant influence on the modifications in the secondary structure of the protein. J. Min et al. studied the interaction of cinnamic acid and its hydroxyl derivatives with human serum albumin. According to their findings, the drug–HSA interaction led the protein polypeptide chain to reorganize and the protein's secondary structure to alter. When HSA interacted with caffeic acid, p-coumaric acid, and cinnamic acid, the alpha-helix shape was reduced by 9%, 5%, and 3%, respectively, as the number of hydroxyl groups increased [3]. Finally, in SML-ANPS, the changes of the previous mentioned peaks shapes intensity demonstrated that the secondary structure of the HAS was changed by the interaction of GLU with HAS.

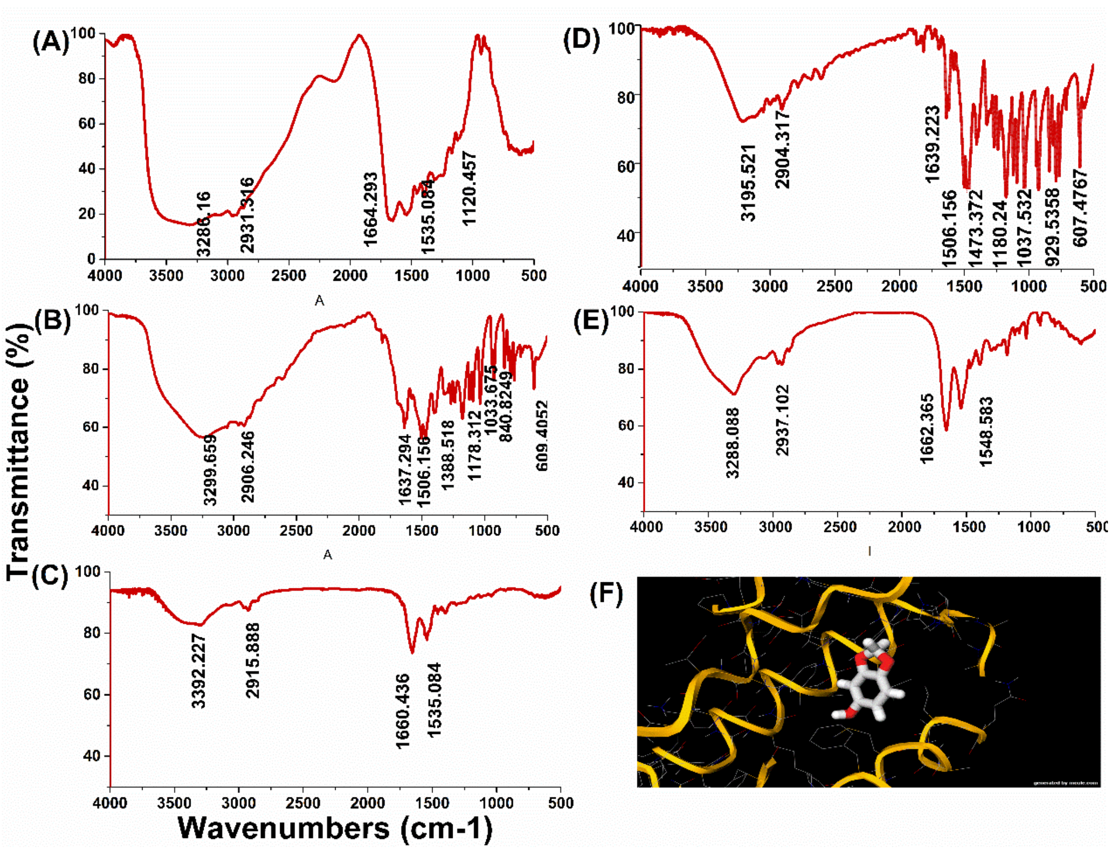

Figure S2. FTIR spectra of HSA (A) SML (B) SML/HSA physical mixture (C) SML/HSA mixture after incubation (D), and SML-ANPS (E) together with the successful docking of sesamol on HSA (F).

Samples collection and tissue homogenate preparations

We collected blood samples from the examined animals via the medial canthus of the eye. After centrifugation of the blood samples at 4000 rpm for 15 minutes, serum was pipetted off and stored at -80 °C for subsequent biochemical analysis. Then, by cervical dislocation, the animals were sacrificed, tissue samples were dissected and instantly rinsed with sterile ice-cold physiological saline. The first set was frozen in liquid nitrogen and kept at -80 °C until homogenisation with 0.115 M ice-cold phosphate-buffered saline (1:5 w/v). A 10% (w/v) homogenate was centrifuged at 10,000 rpm for 15 minutes at − 4 °C. The supernatant was pipetted off and separated into aliquots before being kept at -80 ° C for inflammatory markers assessment. Finally, a portion of each tissue specimen was stored in 10% neutral buffered formalin for future histological studies.

Table S1. Physicochemical characteristics of SML-ANPs.

Run	D.A.	PH	HSA (mg)	Particle size (nm)	PDI	Zeta potential (ζ)	EE (%)	Yield (%)	DL (µg /mg NPs)
**1**	**-1**	**-1**	**-1**	269.14±6.64	0.22±0.11	-21.2±2.6	39.7±1.2	46.15±4.37	79.7±8.9
**2**	**-1**	**-1**	**0**	304.47±3.80	0.29±0.08	-22.87±1.5	41.7±3.37	66.55±1.92	58.9±4.8
**3**	**-1**	**-1**	**+1**	367.11±4.57	0.11±0.05	-23.25±1.8	59.56±0.86	86.3±2.99	33.38±0.7
**4**	**-1**	**0**	**-1**	120.39±1.13	0.19±0.22	-33.75±1.76	59.06±1.3	44.75±2.33	116.69±3.24
**5**	**-1**	**0**	**0**	148.36±5.08	0.13±0.07	-36.62±0.62	62.4±0.79	69.65±3.13	82.32±3.9
**6**	**-1**	**0**	**+1**	168.02±4.8	0.16±0.04	-36.48±1.97	66.16±2.9	85.85±2.27	37.11±1.6
**7**	**-1**	**+1**	**-1**	195.8±3.61	0.29±0.13	-40.28±1.76	17.3±0.81	42.57±1.83	39.12±3.1
**8**	**-1**	**+1**	**0**	223.43±4.3	0.16±0.08	-41.42±1.39	22.5±3.3	69.27±2.67	31.38±3.6
**9**	**-1**	**+1**	**+1**	242.58±7.8	0.19±0.05	-42.07±2.01	29.5±0.95	72.52±2.51	19.96±1.1
**10**	**+1**	**-1**	**-1**	255.17±3.48	0.182±0.09	-15.03±1.56	62.3±1.4	57.88±3.66	97.4±65
**11**	**+1**	**-1**	**0**	287.72±2.26	0.23±0.075	-15.77±1.39	66.13±0.95	75.36±0.84	80.67±0.3
**12**	**+1**	**-1**	**+1**	328.14±13.03	0.21±0.12	-16.75±1.06	69.56±1.4	95.58±1.19	35.12±0.9
**13**	**+1**	**0**	**-1**	111.46±4.47	0.12±0.059	-23.62±0.67	73.26±2.5	55.82±3.32	116.12±2.6
**14**	**+1**	**0**	**0**	127.24±2.12	0.07±0.014	-26.2±1.53	87.86±1.0	81.92±1.6	96.89±2.4
**15**	**+1**	**0**	**+1**	159.99±0.68	0.12±0.04	-29.6±1.71	91.26±3.1	93.2±0.61	48.13±1.4
**16**	**+1**	**+1**	**-1**	179.74±4.2	0.11±0.09	-35.85±1.17	20.03±0.58	55.61±4.79	34.94±3.12
**17**	**+1**	**+1**	**0**	209.97±7.5	0.16±0.07	-37.58±1.07	23.23±1.5	79.62±4.94	28.35±0.3
**18**	**+1**	**+1**	**+1**	233.43±0.417	0.11±0.04	-38.07±1.34	27±1.38	89.86±0.47	14.8±0.7

Abbreviations: D.A: Desolvating agent; DL: Drug loading; EE%: Percent of entrapment efficiency; HSA: Humane serum albumin; SML-ANPS: Sesamol-loaded albumin nanoparticles; PDI: Polydispersity index; PS: Particle size; Y%: Percent of yield; ZP: Zeta potential.
Note: All results expressed mean ± SD, n=3.

Table S2. The effect of the factors controlling SML-ANPs preparation and their levels in the D-optimal design, where pH is the most significant factor.					
**Factors/responses**	**PS**	**PDI**	**Zeta**	**%EE**	**%Yield**	**LC**
**PH**	***	Ns	***	***	Ns	***
**HSA concentration**	***	Ns	*	*	***	**
**D.A**	**	Ns	***	**	***	Ns
**Model**	***	Ns	***	***	***	***

Abbreviations: D.A: Desolvating agent; DL: Drug loading; EE%: Percent of entrapment efficiency; HSA: Humane serum albumin; SML-ANPS: Sesamol-loaded albumin nanoparticles; PDI: Polydispersity index; PS: Particle size; Y%: Percent of yield; and ZP: Zeta potential.
Note: For all results, the differences were considered significant when P<0.05, highly significant when p < 0.001 and extremely significant when p < 0.0001 presented by *, ** and ***, respectively.

Table S3. Release kinetics of SML-ANPs (F14).

Model	Zero order	Fist order	Huguchi	Hixson	Korsmeyers- peppas
**R^2 value**	0.879	0.953	0.971	0.932	0.985

Where, the diffusion exponents value (n) of Korsmeyers- peppas model was 0.347

Abbreviation: R^2: Correlation Coefficient Value and SML-ANPS: Sesamol-loaded albumin nanoparticles.

Table S4. Hepatoprotective effect of free SML and SML-ANPs on rat hepatocytes measured by the MTS assay.

Treatment	Tested Conc.	Viability %	Hepatoprotective %
**SML + 1µM Dox**	500µg/ml	55.28±3.55	30.11±4.57
	100µg/ml	41.90±3.64	16.72±4.41
**SML-ANPS + 1µM Dox**	500µg/ml	66.85±5.07	41.67±4.98
	100µg/ml	48.38±7.03	23.21±6.12
**Doxorubicin**	1µM	25.18±1.37	-

Abbreviation: SML: sesamol, SML-ANPS: Sesamol-loaded albumin nanoparticles and DOX: doxorubicin. Note: All results presented as %hepatoprotection and expressed as the mean ± SD (n in each group = 4).

**Table S5. The histopathological scores of the liver, kidney, heart, and testis in each groups [a].**					
**Organ**	**Lesion**	**Groups b**			
		**Control GP**	**DOX GP**	**SML GP**	**SML-ANPs GP**
**Liver**	**1. Atrophy of the hepatocytes**	**0**	**3**	**2**	**1**
	**2. Cytoplasmic vacuolation**	**0**	**2.4**	**2**	**1**
	**3. Hepatocyte necrosis**	**0**	**2.6**	**1**	**1**
	**4. Lymphoid cell aggregation**	**0**	**3**	**2**	**1**
**Kidney**	**1. Glomeruli necrosis and atrophy**	**0**	**3**	**1**	**1**
	**2. Hyaline cast deposition**	**0**	**3**	**2**	**1**
	**3. Renal tubular necrosis**	**0**	**3**	**2**	**1**
	**4. Lymphoid cell aggregation**	**0**	**2**	**1**	**1**
**Heart**	**1. Necrosis of myocardial cells**	**0**	**2**	**1**	**0**
	**2.Lymphoid cell infiltration**	**0**	**2**	**1**	**1**
	**3. Cytoplasmic vacuolation**	**0**	**2**	**1**	**0**
	**4. Interstitial edema**	**0**	**1**	**0**	**0**
**Testis**	**1. Damage to seminiferous tubules**	**0**	**3**	**2**	**1**
	**2. Cytoplasmic vacuolation**	**0**	**2**	**0**	**0**
	**3. Intratubular multinucleated spermatids**	**0**	**2**	**0**	**0**
	**4. Hyaline degeneration**	**0**	**2**	**2**	**1**

[a]0 = no damage; 1 = (< 25% damage) focal, slight changes; 2 = (25–50% damage) multifocal, significant changes; 3 = (> 50% damage) common widespread changes. Abbreviations: DOX GP: doxorubicin-treated group; SML GP: doxorubicin + sesamol treated group; SML-ANPs GP: doxorubicin + albumin nanoparticles treated group.

Evaluation of Cytotoxic Effects on rat hepatocytes

Rat Hepatocyte Isolation:

Sprague-Dawley male rats (200-250 g) were obtained from the Animals House in Faculty of Science at Al-Azhar University, Cairo, Egypt. Hepatocyte isolation was performed according to the collagenase perfusion procedure which was described by [4]. Hepatocytes (1×10^6 cells/ml) were placed into Krebs-Henseleit buffer (pH: 7.4) containing 12.5 mM HEPES (Sigma-Aldrich, UK) and kept at 37 °C with 95% O2 and 5% CO2. Hepatocytes with a viability of more than 90%, which was measured

with Trypan Blue (Sigma-Aldrich, UK), were used in the experiments (Shen *et al.*, 2012).

Cytotoxicity evaluation using viability assay: For cytotoxicity assay, the hepatocytes were grown on RPMI-1640 medium supplemented with 10% inactivated fetal calf serum and 50µg/ml gentamycin., at concentration $2x10^6$ cell/well in Corning® 96-well tissue culture plates, then incubated for 24 hr at 37ºC in a humidified atmosphere with 5% CO_2 were suspended in medium at concentration $2x10^6$ cell/well in Corning® 96-well tissue culture plates. The tested compound was then added into 96-well plates (three replicates) to achieve ten concentrations (2000, 1500, 1000, 750, 500, 100, 50, 25, 10 and 5 µg/ml). Six vehicle controls were run for each 96 well plate as a control. After incubating for 48 h, the numbers of viable cells were determined by the MTT test. Briefly, the media was removed from the 96 well plate and replaced with 100 µl of fresh culture RPMI 1640 medium without phenol red then 10 µl of the 12 mM MTT stock solution (5 mg of MTT in 1 mL of PBS) to each well including the untreated controls. The 96 well plates were then incubated at 37°C and 5% CO_2 for 4 hours. An 85 µl aliquot of the media was removed from the wells, and 50 µl of DMSO was added to each well and mixed thoroughly with the pipette and incubated at 37°C for 10 min. Then, the optical density was measured at 590 nm with the microplate reader (SunRise, TECAN, Inc, USA) to determine the number of viable cells and the percentage of viability was calculated as [(ODt/ODc)]x100% where ODt is the mean optical density of wells treated with the tested sample and ODc is the mean optical density of untreated cells. The relation between surviving cells and drug concentration is plotted to get the survival curve of hepatocytes after treatment with the specified formula .The 50% cell cytotoxic concentration (CC_{50}), the concentration required to cause toxic effects in 50% of intact cells, was estimated from graphic plots of the dose response curve for each conc. using Graphpad Prism software (San Diego, CA. USA) (Mosmann, 1983).

Evaluation of sesamol cytotoxicity against Rat hepatocytes cell line after 48 h.

Inhibitory activity against rat hepatocytes was detected using MTT assay under these experimental conditions for 48 h incubation with CC_{50} =728.60 ± 60.83 µg/ml.

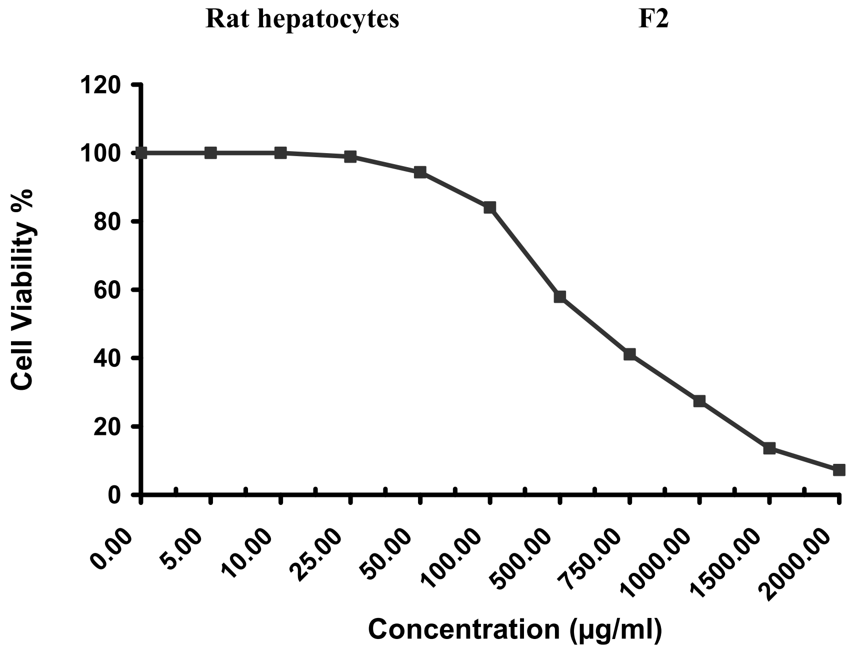

Figure S3. Dose-response curve for sesamol effects on cell viability of rat hepatocytes to calculate the CC 50. The values are presented as mean ± SD (n in each group = 3).

Table S6. Evaluation of sesamol cytotoxicity against Rat hepatocytes cell line after 48 h.

Sample conc. (µg/ml)	Viability% (3 Replicates)			Mean Viability %	Inhibitor y %	S.D. (±)
	1st	2nd	3rd			
2000	7.64	6.49	7.64	7.26	92.74	0.66
1500	14.51	15.38	10.89	13.59	86.41	2.38
1000	27.82	29.06	25.23	27.37	72.63	1.95
750	39.87	42.65	40.72	41.08	58.92	1.42
500	58.04	61.37	54.18	57.86	42.14	3.60
100	83.95	81.98	86.29	84.07	15.93	2.16
50	95.71	93.2	94.12	94.34	5.66	1.27
25	99.43	98.76	98.46	98.88	1.12	0.50
10	100	100	100	100	0	
5	100	100	100	100	0	
0	100	100	100	100	0	

Note: All results expressed mean ± SD, n in each group = 3.

Refrences

1. Hassanzadeh, P., F. Atyabi, R. Dinarvand, A.-R. Dehpour, M. Azhdarzadeh and M. Dinarvand, *Application of nanostructured lipid carriers: the prolonged protective effects for sesamol in in vitro and in vivo models of ischemic stroke via activation of PI3K signalling pathway.* DARU Journal of Pharmaceutical Sciences, 2017. **25**(1): p. 25 DOI: 10.1186/s40199-017-0191-z.
2. Li, J., T. Chen, F. Deng, J. Wan, Y. Tang, P. Yuan and L. Zhang, *Synthesis, characterization, and in vitro evaluation of curcumin-loaded albumin nanoparticles surface-functionalized with glycyrrhetinic acid.* International journal of nanomedicine, 2015. **10**: p. 5475 DOI: doi.org/10.2147/IJN.S88253.
3. Min, J., X. Meng-Xia, Z. Dong, L. Yuan, L. Xiao-Yu and C. Xing, *Spectroscopic studies on the interaction of cinnamic acid and its hydroxyl derivatives with human serum albumin.* Journal of Molecular Structure, 2004. **692**(1-3): p. 71-80 DOI: 10.1016/j.molstruc.2004.01.003.

4. Reese, J.A. and J.L. Byard, *Isolation and culture of adult hepatocytes from liver biopsies.* In Vitro, 1981. **17**(11): p. 935 DOI: https://doi.org/10.1007/BF02618417.
